# 67. A rare case of aortitis as the presenting feature of haematological malignancy

**DOI:** 10.1093/rap/rky034.030

**Published:** 2018-09-20

**Authors:** Laura Spooner, Gurdeep Dulay, Nicholas Fuggle, Jessica Woodley

**Affiliations:** Rheumatology, Queen Alexandra Hospital, Portsmouth, UNITED KINGDOM

**Introduction:** Large vessel vasculitis is a rare condition and normally presents in the context of giant cell or Takayasu’s arteritis. While autoimmune conditions are well recognised in myelodysplasia, large vessel vasculitis is very rare. We describe an unusual case of a patient with no previous rheumatological or haematological diagnosis presenting with aortitis associated with new myelodysplastic syndrome, likely to represent chronic myelomonocytic leukaemia. This has implications for the management of patients with large vessel vasculitis (or other autoimmune conditions) who do not respond fully to immunosuppression, or who have atypical features.

**Case description:** A 61 year old man presented to AMU with two weeks of fever, malaise, night sweats and weight loss. He reported pleuritic chest pain, shortness of breath and diarrhoea. There were no other significant symptoms. Aside from hypertension and type II diabetes mellitus, he was generally well. He had not received previous immunosuppressive drugs. On initial examination he was pale and appeared systemically unwell. He had marked peripheral oedema and a purpuric rash on both lower legs. The remainder of the systemic examination was unremarkable. Initial investigations showed Hb 78 (130-180g/l), wcc 11.7 (4.5-11 x 10^9^/l), Plt 187 (150 - 450 x 10^9^/l), CRP 384 (<11mg/l), ESR 87 (<10mm/h) and ferritin 3000 (15-300ng/ml). Of particular note, his monocyte count was significantly elevated at 4.4 (0.2-0.8 x 10^9^/l). Albumin was 14 (35-50g/l) and urine protein:creatinine ratio was elevated at 208 (<30mg/mmol). Creatinine, initial blood film and chest x-ray were normal. He was treated for presumed underlying infection with broad spectrum IV antibiotics (no clear source identified), but unfortunately did not improve clinically or biochemically. His CRP remained above 300 despite a number of days of treatment. A subsequent CT abdomen/pelvis showed pan-aortitis of the ascending aorta extending into the neck and descending aorta. There was no stenosis, dissection or rupture. He received initial treatment for large vessel vasculitis with 3 x IV methylprednisolone 500mg. This was followed by 40mg prednisolone daily with bone protection and a PPI. Crucially, although there was a degree of transient clinical improvement, his abnormal blood counts persisted despite IV methylprednisolone. This is unusual for typical cases of large vessel vasculitis. He developed progressively worsening cytopenias with Hb 68 (130-180g/l), Plt 31 (150-450 x 10^9^/l) and lymphocytes 0.3 (1.5-4.5 x 10^9^/l). Monocytes remained high at 4.5 (0.2-0.8 x 10^9^/l). This would not be expected in isolated large vessel vasculitis, but could be accounted for by an underlying malignancy or auto-inflammatory process. This was a cause for concern given the likely need for ongoing immunosuppression. Of note, a full infection, autoimmune and myeloma screens were negative. These included rheumatoid factor, anti-CCP antibody, ANA, complement, cryoglobulins, immunoglobulins and IgG subclasses. Multiple blood, urine and stool cultures, HIV, hepatitis B and C serology, ASO, mycoplasma, syphilis, CMV, EBV and influenza PCR were also negative. B_12_/folate and LDH were normal, although clotting was deranged with INR 1.4 (0.8-1.2) and fibrinogen 7.8(1.5-4.5 g/l). Haptoglobin was raised at 5.32 (0.3-2.0g/l). A transoesophageal echo was also normal. A bone marrow biopsy showed marked dysplastic changes with blast count 8% and trisomy 8, consistent with MDS RAEB1. The monocytosis may be significant and is suggestive of CMML, a subset of MDS. Only four previous case reports have described aortitis as a complication of CMML. He has now been referred for bone marrow transplant and has started azacitidine treatment for MDS. A PET CT shows no active vasculitis, lymphadenopathy or other lesion. This suggests his ongoing low cell counts and raised inflammatory markers are related to his underlying haematological malignancy. He is gradually improving clinically and has been discharged. His prognosis is likely to be less than five years from time of diagnosis.

**Discussion:** Ten to twenty percent of patients with MDS present with systemic inflammatory or autoimmune conditions. However, most cases of vasculitis linked to MDS affect small vessels. Large vessel vasculitis is rare (with only four reported cases linked to CMML). In most of these cases the aortitis has preceded transformation to acute myeloid leukaemia. While our patient’s aortitis was recognised early and treated promptly, his presentation had some atypical features which can indicate underlying malignancy. In particular, his lack of full response to high dose steroids and the continued presence of cytopenias in multiple cell lines would not be expected in large vessel vasculitis alone and should prompt the search for an underlying cause. A ferritin count of 3000 is also another significant pointer towards possible underlying haematological pathology. In this case the rapid clinical onset of both MDS and aortitis suggests that the large vessel vasculitis is likely to be a paraneoplastic phenomenon. Treating vasculitis or other autoimmune disease in the context of MDS is challenging. Patients are likely to be at high risk of infection and have limited marrow reserve. Outstanding questions remain regarding the effect of immunosuppression (for treatment of vasculitis) on the response to an underlying malignancy. Conversely, it is not fully understood whether treating the underlying malignancy also treats the autoimmune disease, as might be expected if caused by a paraneoplastic phenomenon. Studies show that azacitidine can improve symptoms of both MDS and systemic inflammatory/autoimmune disease, and reduce steroid dependence. However, allogeneic bone marrow transplant is the only curative treatment for MDS.

**Key Learning Points:** Large vessel vasculitis (or other autoimmune conditions) may be the first presenting symptom of a haematological malignancy. This should be kept in mind when managing patients with large vessel vasculitis, especially if they present with atypical features or do not respond conventionally and fully to standard dose immunosuppression. Urgent haematology review with a view to undertaking a bone marrow examination is of vital importance. If MDS is diagnosed, close monitoring for transformation to AML should be undertaken. Prompt treatment of the underlying condition may improve prognosis.

**Disclosure: L. Spooner:** None. **G. Dulay:** Honoraria; Amgen, Lilly, Sandoz. Other; Funding for educational events and activities from Pfizer, UCB, Abbvie, Roche/Chugai. **N. Fuggle:** None. **J. Woodley:** None.

